# Hemorrhagic complications in a pelvic kidney: the role of interventional radiology: A case report

**DOI:** 10.1016/j.radcr.2025.10.055

**Published:** 2025-11-08

**Authors:** Matteo Haupt, Thomas Jansen, Ole Jeske, Martin H. Maurer, Rohit Philip Thomas

**Affiliations:** a Department of Diagnostic and Interventional Radiology, Carl von Ossietzky Universität Oldenburg, Oldenburg, Germany; b Department of Urology, Carl von Ossietzky Universität Oldenburg, Oldenburg, Germany

**Keywords:** Pelvic kidney, Embolization, Hemorrhage control, Interventional radiology

## Abstract

Pelvic kidney is a rare congenital anomaly characterized by aberrant location and complex vascular anatomy, predisposing it to complications such as trauma and hemorrhage. A 57-year-old man with a known right-sided pelvic kidney presented with acute macroscopic hematuria. Computed tomography revealed a large pericapsular hematoma with aberrant arterial supply and suspected active extravasation. A multidisciplinary approach was adopted, beginning with endovascular embolization as a preoperative measure to control bleeding, followed by transabdominal nephrectomy as definitive treatment. The combined approach ensured effective hemorrhage control and minimized surgical risk. This case highlights the importance of preoperative embolization as a safe and valuable adjunct in the management of hemorrhage in anatomically complex renal anomalies such as pelvic kidneys and underscores the role of multidisciplinary collaboration in achieving optimal outcomes.

## Background

The kidneys are retroperitoneal organs encased in adipose tissue, located between the 12th thoracic vertebra and the 3rd lumbar vertebra. Pelvic ectopia occurs in approximately 1 in 2500 births and is of both surgical and radiological importance [1]. Pelvic kidneys are often asymptomatic and typically discovered incidentally. However, due to their abnormal rotation, shape, and vasculature, pelvic kidneys predispose patients to various complications, including urinary tract infections, renal calculi, uretero-pelvic junction obstructions, and vesicoureteral reflux. The ectopic position of these kidneys also increases their vulnerability to physical trauma [2].

The vasculature of pelvic kidneys is known to be complex and highly variable, primarily due to the retention of fetal blood supply. Numerous variations at autopsy, with arterial supply ranging from single to double or triple arteries, originating from the aortic bifurcation, iliac artery, or hypogastric artery are described [3].

We report a patient with a known pelvic kidney who presented with severe macroscopic hematuria. Diagnostic workup revealed a large hematoma and an indistinguishable hematoma/kidney complex, as well as vascular pathology suggesting acute arterial hemorrhage or an intraparenchymal pseudoaneurysms of the abnormal renal arteries. After multidisciplinary discussion with urology, an attempt to retain the pelvic kidney was discussed. Consequently the patient received a ureteral stent with conservative measures including pain management. Endovascular intervention with embolization was discussed to control the bleeding in the acute setting and to facilitate a safer resection with lesser bleeding complications, if at all needed. The next day patient showed clinical and laboratory signs of further bleeding. Endovascular intervention was performed with coiling of aberrant vascular supplies of the pelvic kidney, after which nephrectomy was performed uneventfully with lesser bleeding complications.

This case highlights the challenges arising from abnormal anatomy, particularly in aberrant vascular supply of pelvic kidneys, which impact both diagnostic and interventional approaches in the treatment decisions. It emphasizes the importance of interdisciplinary management and the benefits of preoperative vascular interventions in achieving successful clinical outcomes.

## Case presentation

A 57-year-old male patient was referred to the hospital by his general practitioner due to significant macroscopic hematuria. The patient had a known history of a right-sided pelvic kidney with associated functional impairment and hydronephrosis. Dysuria was not reported.

On admission, physical examination revealed mild tenderness in the right lower abdomen without guarding or palpable pathological masses. Subsequent sonographic evaluation confirmed the absence of the right kidney in its usual anatomical position, consistent with the known pelvic kidney. Furthermore, an unclear cystic structure, likely representing the pelvic kidney, was visualized in the lower abdomen. Additional suspicious inhomogeneities raised the possibility of a hematoma. Laboratory investigations revealed a mildly decreased hemoglobin level of 10.9 g/dL, and urinalysis was markedly positive for erythrocytes.

During the same day, cystoscopy was performed. The urethra appeared unremarkable. Initial panoramic cystoscopy of the bladder was limited due to bloody urine. The ureteral orifices were orthotopically located on both sides. Significant bleeding was observed from the right ureteral orifice. Periprocedural sonographic evaluation of the bladder revealed a mass next to the bladder on the right side. Differential diagnoses included a tumor, hematoma, the previously identified right-sided pelvic kidney, or a ureteral tumor. A CT scan was ordered for further diagnostic clarification.

**Fig. 1:**
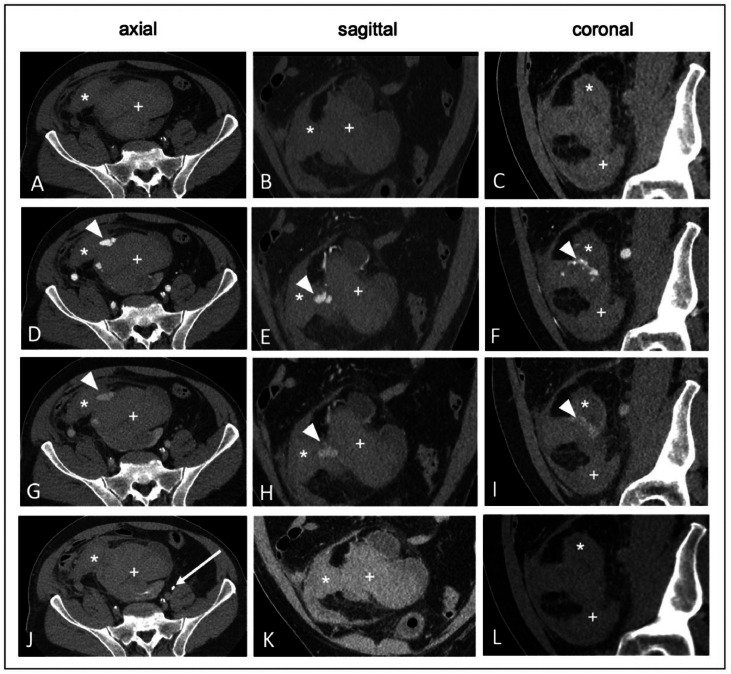
CT images of the pelvis, including one native (noncontrast) phase (A-C) and three contrast-enhanced phases: arterial (D-F), venous (G-I), and urographic (J-L). Key findings are highlighted: ► indicates findings suspicious for vascular pathology (eg, contrast extravasation or aneurysm); * denotes hematoma; + represents the pelvic kidney/hematoma complex; and an arrow in (J) points to the contrast enhancement of the left ureter.

The CT scan revealed findings consistent with a large hematoma involving the right pelvic kidney, extending from the mid-abdomen to the pelvic region (Fig. 1). Minimal residual renal parenchyma was identified at the level of the renal hilum. The hematoma extended intraperitoneally into the right lower abdomen. The arterial supply to the right kidney was observed to originate from the aortic bifurcation. Furthermore, there was a suspicion of arterial extravasation suggestive of acute arterial bleeding, although the contrast behavior was not entirely phase-typical (Fig. 1 D-F). Notably, no definitive venous pooling was observed, which, from a differential diagnostic perspective, could suggest a potential early tamponade effect from the hematoma or intraparenchymal pseudoaneurysms. In addition, the urographic phase showed no contrast medium excretion from the right kidney (Fig. 1 J-L), confirming the history of impaired renal function.

After multidisciplinary discussion with urology, an attempt to retain the pelvic kidney was discussed. Consequently the patient received a ureteral stent with conservative measures including pain management. Bloody urine with small clots was noted in the bladder. The patient experienced significant pain relief after urine drainage, with a substantial outflow of blood-tinged urine. Endovascular intervention with embolization was discussed to control the bleeding in the acute setting and to facilitate a safer resection with lesser bleeding complications, if at all needed. The approach was developed through interdisciplinary discussions, with ongoing monitoring of the patient’s hemoglobin levels and regular ultrasound evaluations to guide and adjust the treatment strategy as necessary.

The following day, the patient showed clinical and laboratory signs of ongoing bleeding. An interdisciplinary decision was made to perform a planned nephrectomy after endovascular embolization to reduce bleeding complications. Given the limited function of the right pelvic kidney, nephrectomy was justified.

**Fig. 2:**
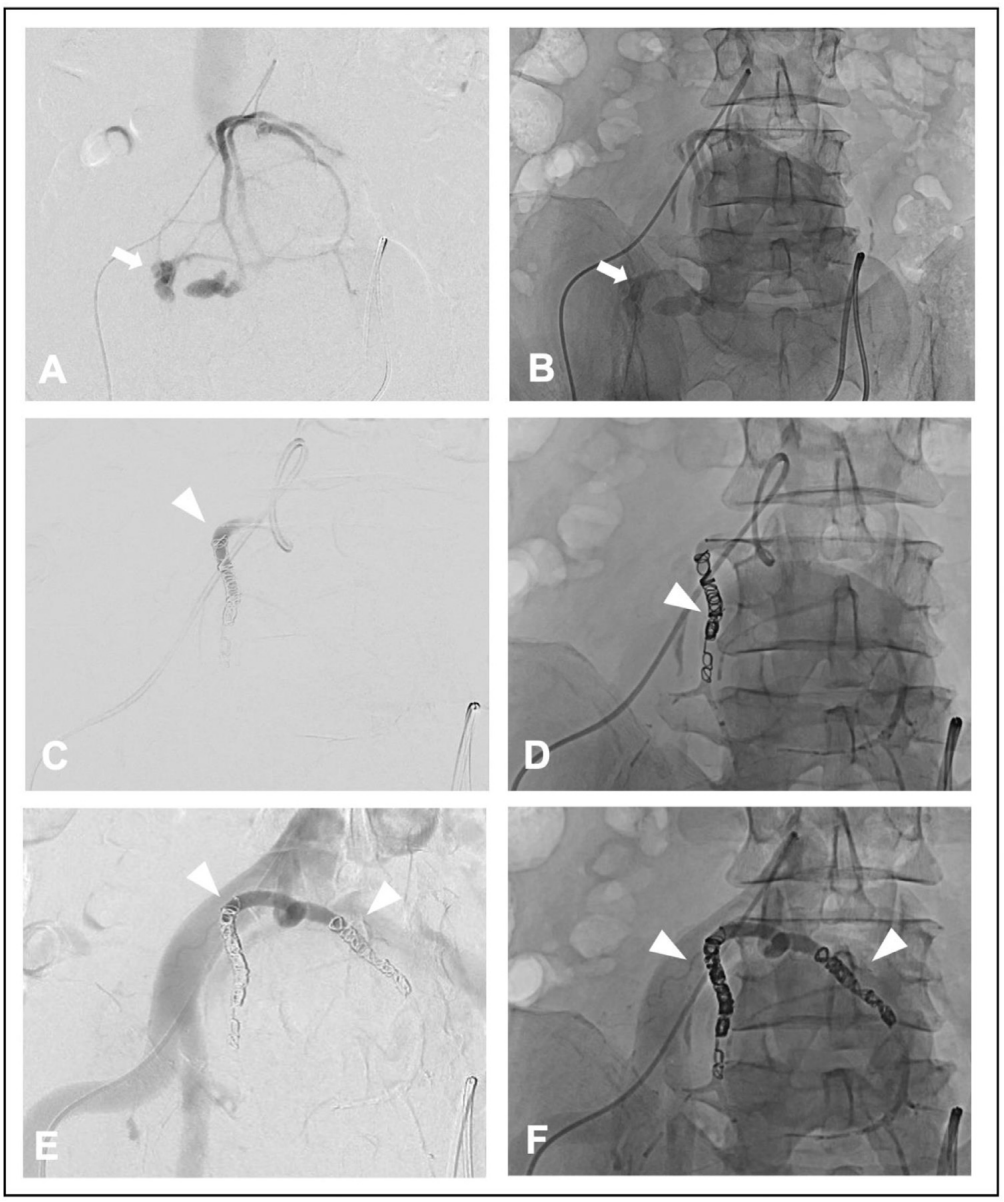
Images of endovascular embolization of the arterial supply to the right-sided pelvic kidney with digital subtraction angiography (A, C, E) and nonsubtracted images (B, D, F). The arrows indicate enlarged and altered vessels, including multiple pseudoaneurysms. ► marks the placed metal coils.

As part of the plan, preoperative embolization of the right pelvic kidney was performed as an urgent emergency intervention. Under sterile conditions with local anesthesia, a retrograde transfemoral puncture was conducted on the right side, and a 5F Prelude 11 cm sheath (Merit Medical Inc, USA) was introduced. Using a 4 F Sidewinder I catheter (100 cm; Terumo, Tokyo, Japan), the aortic bifurcation was visualized and the origin of the aberrant renal artery was identified. Two major branches, each approximately 5 mm in diameter which supplied the pelvic kidney were identified, arising from a common stem directly originating from the ventral aortic bifurcation (Fig. 2 A-B). Two pseudoaneurysms were also identified on the right side, which correlated with the hyperdensities seen in the CT Scan. The left-sided branch appeared normal. No active extravasation of contrast agent was identified. An angled guidewire (180 cm; Terumo, Tokyo, Japan) was used for navigation. A coaxial Progreat microcatheter (2.4 F, 130 cm and 150 cm; Terumo, Tokyo, Japan) within the Sidewinder catheter facilitated deep probing of the vessels. Partial nephrectomy was considered infeasible due to the extent of vascular abnormalities, leading to the decision to embolize all renal vessels of the right kidney and nephrectomy.

The procedure began with deep probing of the left-sided portion of the pelvic kidney and its vessels. After documenting the correct catheter positioning, embolization was carried out using a mixture of 0.5 ml Glubran (GEM, Italy) and 1.0 ml Lipiodol Ultra Fluid (Guerbet, Villepinte, France) in a 1:2 ratio for permanent embolization. Postembolization imaging showed excellent results, with occlusion of the left-sided vessels and the pseudoaneurysms on the right side. Subsequently, selective probing of the right main branch was performed, followed by embolization using Concerto detachable coils (5/20 cm, 6/20 cm, 2 × 7/30 cm, and 8/30 cm; Medtronic, Germany) (Fig. 2 C-D). Additional embolization of the left-sided main trunk was also completed with Concerto coils. A total of five coils were deployed. The patient complained about slight pain on the right lower abdomen, which could be managed with pain medications. A 5 F MYNXGRIP closure device (Cordis Medical, CA, USA) was used to seal the puncture site. Postprocedural imaging demonstrated excellent results, with no evidence of complications and nearly complete devascularization of the pelvic kidney.

**Fig. 3:**
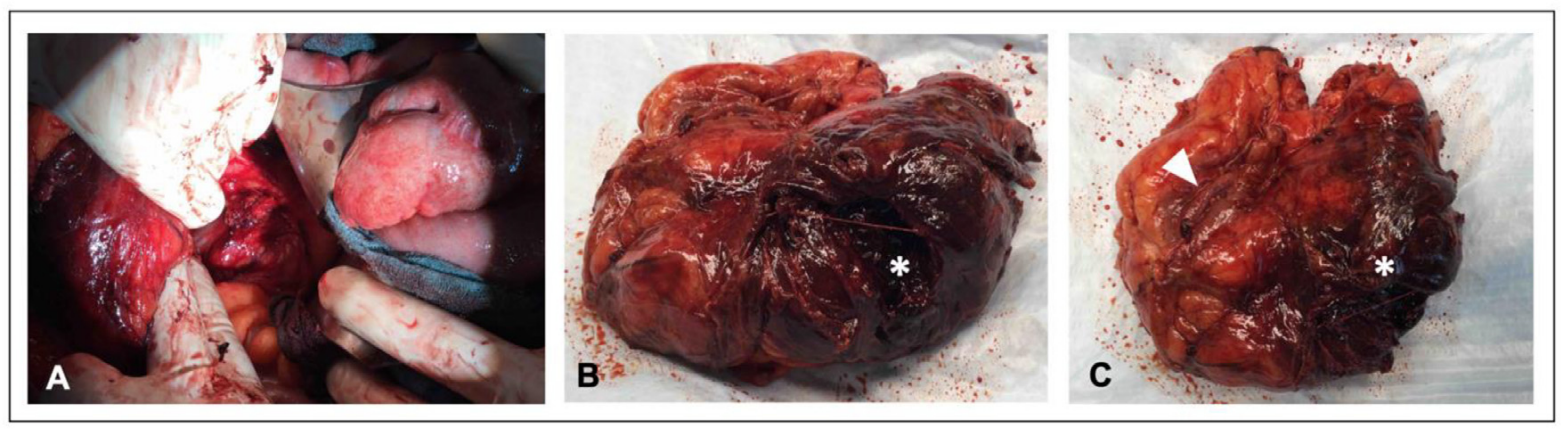
Images documenting the nephrectomy procedure and the removed kidney. (A): Intraoperative photograph showing the surgical site. (B, C): Photographs of the removed kidney. * marks the hematoma identified in the CT scan, and ► indicates part of the metal coils from the endovascular embolization.

Subsequently, on the same day, a transabdominal nephrectomy was performed (Fig. 3 A-C), which included drainage of a large hematoma, yielding approximately 800 ml of blood. The patient, in stable condition, was subsequently transferred to intensive care for further management.

## Discussion

Managing vascular complications in a pelvic kidney is challenging due to its ectopic location and complex vasculature. In this case, macroscopic hematuria and vascular abnormalities required a multidisciplinary approach to ensure effective management.

This constellation also fulfills the criteria of Wunderlich syndrome, defined as spontaneous, nontraumatic renal hemorrhage into the subcapsular and/or perirenal space and often associated with Lenk’s triad of acute flank pain, palpable mass, and hematuria [4,5]. Our patient had hematuria and abdominal pain, and imaging confirmed a large pericapsular hematoma, consistent with this definition. A palpable mass, however, was not detected on clinical examination, most likely due to the ectopic pelvic position of the kidney. Nevertheless, the hemorrhage occurred in the context of an ectopic pelvic kidney, making this an unusual anatomical variant that has rarely been described.

Imaging revealed abnormal vascular supply to the pelvic kidney, consistent with the variability described in the literature [3]. Endovascular embolization via a retrograde transfemoral approach successfully controlled the bleeding and reduced surgical risks of bleeding during resection. Postembolization imaging confirmed the occlusion of pathological vessels, enabling a safer nephrectomy. Partial nephrectomy was not feasible due to the extent of vascular abnormalities, necessitating total nephrectomy. Because nephrectomy was planned, permanent embolization materials (NBCA–Lipiodol and coils) were used to achieve complete devascularization and reduce bleeding risk, whereas temporary agents such as particles or gelfoam are typically reserved for organ-preserving procedures.

Preoperative embolization in renal surgery is well-documented in reducing blood loss during nephrectomy for renal cell carcinoma [6,7]. Reports of interventional radiology management in pelvic kidneys are very limited, with most literature focusing on their anatomical variability and surgical implications [1,2]. Only rare case descriptions exist, such as emergent embolization of a pelvic kidney with metastatic renal cell carcinoma [8]. The scarcity of published cases underlines the clinical relevance of our report. Without embolization, nephrectomy in this setting would likely have been associated with uncontrolled hemorrhage, higher transfusion requirements, and increased perioperative morbidity. The benefits seen in renal cell carcinoma cases, such as reduced blood loss and fewer complications, align with the successful outcomes observed in this case. This demonstrates that embolization can be an effective intervention not only in oncological procedures but also in managing vascular complications in anatomically atypical kidneys.

In this case, embolization was not intended as a definitive therapy but rather as a preoperative adjunct to minimize bleeding risk during surgery. Because the pelvic kidney was nonfunctional and exhibited extensive vascular abnormalities, nephrectomy remained necessary. This underlines that IR can serve both as a potential standalone therapy for hemorrhage control in selected cases and, as in our patient, as a valuable step to ensure safe surgical resection.

## Conclusion

This case highlights the challenges of managing vascular complications in a pelvic kidney, emphasizing the importance of tailored treatment strategies and multidisciplinary approaches. Preoperative embolization was essential in controlling bleeding and facilitating a safer nephrectomy. Embolization is a well-established technique in the context of renal procedures, particularly in oncological settings such as preoperative management to reduce blood loss during nephrectomy for renal cell carcinoma. In the present case, it proved effective for achieving hemorrhage control and enabling safe resection in the anatomically complex setting of a pelvic kidney.

## Patient consent

Written informed consent was obtained from the patient for publication of this case report and any accompanying images.
